# Assessing brain and biological aging trajectories associated with Alzheimer’s disease

**DOI:** 10.3389/fnins.2022.1036102

**Published:** 2022-10-28

**Authors:** Winnie S. Liang, Laura H. Goetz, Nicholas J. Schork

**Affiliations:** ^1^NetBio, Inc., Los Angeles, CA, United States; ^2^Translational Genomics Research Institute, Phoenix, AZ, United States

**Keywords:** brain age, biological age, epigenetic clock, Alzheimer’s disease, epigenetic age, brain age gap, aging biomarkers

## Abstract

The development of effective treatments to prevent and slow Alzheimer’s disease (AD) pathogenesis is needed in order to tackle the steady increase in the global prevalence of AD. This challenge is complicated by the need to identify key health shifts that precede the onset of AD and cognitive decline as these represent windows of opportunity for intervening and preventing disease. Such shifts may be captured through the measurement of biomarkers that reflect the health of the individual, in particular those that reflect brain age and biological age. Brain age biomarkers provide a composite view of the health of the brain based on neuroanatomical analyses, while biological age biomarkers, which encompass the epigenetic clock, provide a measurement of the overall health state of an individual based on DNA methylation analysis. Acceleration of brain and biological ages is associated with changes in cognitive function, as well as neuropathological markers of AD. In this mini-review, we discuss brain age and biological age research in the context of cognitive decline and AD. While more research is needed, studies show that brain and biological aging trajectories are variable across individuals and that such trajectories are non-linear at older ages. Longitudinal monitoring of these biomarkers may be valuable for enabling earlier identification of divergent pathological trajectories toward AD and providing insight into points for intervention.

## Introduction

As of 2022, an estimated 5 million Americans are living with mild cognitive impairment (MCI) and 6.5 million Americans are living with Alzheimer’s Disease [AD] (2022), and this number is predicted to reach 13.8 million by 2060 National Institute on Aging (2022). Identifying strategies for disease prevention, earlier diagnosis, and improved treatment are urgently needed. Notably, changes in the brain, including amyloid deposition, tau phosphorylation, and neuroimaging alterations, which ultimately result in AD, initiate two decades or more before onset of symptoms Alzheimer’s Disease [AD] (2022). Such early changes highlight valuable opportunities to monitor brain and overall health shifts that may precede symptom, or disease, onset, and which may be considered in combination with well-established risk factors, such as age (Hebert et al., 2010), APOEε4 carrier status (Saunders et al., 1993; Farrer et al., 1997), and family history (Fratiglioni et al., 1993; Green et al., 2002). While effective treatments still need to be developed, assessing aging trajectories will enable the implementation of interventions that may help slow disease pathogenesis. In this mini-review, we discuss biomarkers of brain aging and biological aging in the context of cognitive decline and AD.

## Measuring brain age using neuroanatomical changes

One widely adopted neuroimaging approach to assessing brain health is determination of the brain age gap, or predicted age difference between chronologic and brain age, as a biomarker of brain aging and health. The two most widely used methods include the brainAGE (Brain Age Gap Estimation) method (Franke and Gaser, 2019) and that of Cole et al. (2015). These methods primarily employ structural MRI (magnetic resonance imaging) and machine-learning algorithms (Beck et al., 2022), to evaluate neuroanatomical changes in order to estimate an individual’s brain age and its divergence from chronological age (Cole et al., 2015; Franke and Gaser, 2019). A positive brain age gap thus reflects increased brain aging (e.g., higher brain age as compared to chronological age), whereas a negative value reflects slower aging, and in longitudinal analyses, positive values indicate accelerated brain aging, while negative values indicate decelerated aging (Franke and Gaser, 2019). Aging-related structural brain changes that may influence brain age include atrophy (Fjell et al., 2014; Storsve et al., 2014; Grajauskas et al., 2019), loss of white and gray matter (Coupé et al., 2017), and perturbations to functional connectivity (Bennett and Madden, 2014; Damoiseaux, 2017) with increased gray matter atrophy and accelerated changes characterizing progressive MCI and AD (Desikan et al., 2008; Driscoll et al., 2009; Sluimer et al., 2009; Anderson et al., 2012).

## Brain age in the context of cognitive functions and Alzheimer’s disease

The brain age gap biomarker has demonstrated utility in differentiating and predicting changes associated with AD (Table 1). Elevated brain age is associated with decreased cognitive functions in healthy subjects (Richard et al., 2018; Boyle et al., 2021; Elliott et al., 2021; Subramaniapillai et al., 2021), MCI subjects (Franke et al., 2012; Kaufmann et al., 2019; Huang et al., 2021), dementia (Kaufmann et al., 2019), and mortality in elderly subjects (Cole et al., 2018). In a study of early AD, brainAGE scores were an average of 10 years higher (*P* < 0.001) in AD subjects (Franke et al., 2012), and across multiple studies, AD subjects showed accelerated brain aging of 5.36–10 years (Franke et al., 2010, Franke et al., 2012; Löwe et al., 2016; Wrigglesworth et al., 2021). In an analysis of MCI and AD subjects with 3 year follow-up, brainAGE showed increasing mean values across: controls (−0.30 years), subjects with stable MCI (−0.48 years), subjects with progressive MCI (6.19 years), and those with AD (6.67 years) (Franke et al., 2012). Acceleration of brain age was observed with progressive MCI subjects demonstrating a 1.05 year increase in brainAGE with each chronological year and AD subjects demonstrating a 1.51 year increase (Franke et al., 2012). BrainAGE values did not change for control and stable MCI subjects during follow-up and brainAGE correlated with clinical severity and cognitive testing at an individual level. Increased brain age gaps in amnestic MCI subjects have also been found and APOEε4 carriers demonstrate higher ages compared to non-carriers (Huang et al., 2021). Further, the combination of brain age gaps with other AD markers, including APOEε4 carrier status, amyloid status, and MMSE (Mini-Mental State Examination), differentiated progressive from stable MCI (Huang et al., 2021). In separate analyses, APOEε4 carrier status was associated with increased brain age acceleration in progressive MCI or AD subjects, and not in controls or subjects with stable MCI.

**TABLE 1:** Human brain age studies on cognitive functions, mild cognitive impairment (MCI), and/or Alzheimer’s disease (AD).

References	Study focus	Cohorts	No. of subjects (female)	Mean age ± SD	Measurements	Primary findings
Franke et al., 2012	To longitudinally evaluate brain age changes in MCI and AD	ADNI	406 (172)	75.43 ± 1.16	T1-weighted MRI	Acceleration of brainAGE was found in AD patients and subjects who converted to AD within 3 years. An additional increase in brainAGE was found at 9 years follow-up. Brain age acceleration was associated with cognitive decline and disease severity.
Gaser et al., 2013	To determine if brainAGE predicts conversion to AD in MCI subjects	ADNI	579 (318)	70.63 ± 5.78	T1-weighted MRI	BrainAGE predicted conversion of MCI to AD within 3 years of follow-up with 81% accuracy. These predictions were more accurate than the use of cognitive measurements and CSF biomarkers. Each additional brainAGE year was associated with a 10% increased risk of developing AD.
Löwe et al., 2016	To examine the impact of APOE genotype on brain aging in AD	ADNI	405 (NA)	75.55 ± 1.15	T1-weighted MRI	BrainAGE was significantly different across controls, MCI, and AD subjects. BrainAGE correlated with neuropsychological scores in APOEe4 carriers and non-carriers. BrainAGE acceleration was observed in progressive MCI and AD subjects and brainAGE correctly predicted conversion of MCI to AD even without APOE genotype.
Boyle et al., 2021	To assess any association between brain age gap and cognitive functions	TILDA; CR/RANN; Dokuz Eylul University	1,359 (855)	40.04 ± 17.78	T1-weighted MRI	Brain age gap was negatively correlated with general cognitive performance (2 cohorts); processing speed, visual attention, and cognitive flexibility (3 cohorts); visual attention and cognitive flexibility (2 cohorts); semantic verbal fluency (2 cohorts)
Elliott et al., 2021	To evaluate brainAGE, pace of biological aging, and cognitive decline in longitudinal data in middle-aged adults	Dunedin Longitudinal Study	1,037 (497)	45.15 ± 0.69	T1-weighted MRI; cognitive function batteries	Subjects with older brainAGE had worse cognitive functions in both childhood and adulthood, along with accelerated pace of biological aging, and older facial appearance.
Gautherot et al., 2021	To evaluate predicted brain age difference in relation to phenotypic heterogeneity and severity in early-onset AD	COMAJ, PPMI, ADNI	232 (125)	58.99 ± 0.35	3D T1-weighted MRI	Early-onset AD subjects had higher brain age differences compared to controls and non-amnestic subjects had greater brain age differences compared to amnestic subjects and controls. Increases in brain age differences correlated with disease severity. Predicted age difference was predictive of low MMSE and high CDR in early-onset AD subjects.% gray matter volume, and not white matter volume, was negatively associated with brain age differences.
Gonneaud et al., 2021	To assess if brain age, determined using resting state functional connectivity (rsFC), predicts chronological age across life and if brain age demonstrates changes in preclinical familial AD, including in relation to beta-amyloid pathology	DIAN, PREVENT-AD, CamCAN, FCP-Cambridge, ADNI, ICBM	1,340 (557)	46.71 ±6.76	T1-weighted MRI; Amyloid-beta (Abeta) PET	Brain aging was accelerated in subjects with pre-clinical AD; this association correlated with subjects with increased amyloid-beta deposition per PET. In subjects at risk for sporadic AD, APOEe4 carrier status and Abeta were not associated with accelerated brain age, but asymptomatic subjects who were close to expected age of symptom onset tended to show accelerated brain aging.
Huang et al., 2021	To determine if brain age captures deviations from healthy aging in amnestic MCI (aMCI) patients and is associated with cognitive deficits	BABRI, ADNI	974 (587)	69.95 ± 5.59	T1-weighted MRI	aMCI patients had higher brain age differences than controls and differences are associated with cognitive impairment. APOEe4 carriers had elevated brain age differences compared to non-carriers. aMCI patients who were amyloid-positive had higher brain age differences than amyloid-negative subjects. Brain age differences along with AD markers differentiated progressive from stable MCI at baseline.
Subramaniapillai et al., 2021	To evaluate if there are sex differences associated with brainAGE in relation to AD risk factors in cognitively healthy adults	DLBS, SALD, MMALS, PREVENT-AD	1,067 (697)	52.02 ± 8.35	T1-weighted MRI	Female APOEe4 carriers with family history of AD had more advanced brain aging than men. In this same sub-cohort, higher BMI was associated with less brain aging and lower BMI was not. In APOEe4 carriers, engaging in physical activity was more beneficial to men’s brain aging.
Millar et al., 2022	To predict brain age in preclinical AD using rsFC	Knight ADRC, WUSTL, DIAN	941 (537)	53.59 ± 18.96	T1-weighted MRI; Abeta PET	Symptomatic AD subjects had significantly increased brain age; preclinical AD subjects and APOEe4 carriers had decreased brain age. The latter findings may be associated with biphasic responses to AD pathogenesis or may reflect dysfunction in functional networks in preclinical AD.

ADNI, Alzheimer’s Disease Neuroimaging Initiative; TILDA, The Irish Longitudinal Study on Aging; CR/RANN, Cognitive Reserve/Reference Ability Neural Network; COMAJ, Cohorte Malade Alzheimer’s Jeunes; PPMI, Parkinson’s Progression Markers Initiative; DIAN, Dominantly Inherited Alzheimer Network; PREVENT-AD, Presymptomatic Evaluation of Experimental or Novel Treatments for AD; CamCAN, Cambridge Centre for Ageing and Neuroscience; FCP-Cambridge, 1000-Functional Connectomes Project-Cambridge; ICBM, International Consortium for Brain Mapping; BABRI, Beijing Aging Brain Rejuvenation Initiative; ADRC, Alzheimer’s Disease Research Center; WUSTL, Washington University in St. Louis; DLBS, Dallas Lifespan Brain Study; SALD, South Asian Lifespan Dataset; MMALS, Montreal Memory and Aging Lifespan Study.

Brain age has also been shown to predict conversion of MCI to AD. Elevated baseline brain age values were associated with an increased risk of conversion from MCI to AD, with more accurate predictions in APOEε4 carriers (Löwe et al., 2016). In a study of 195 MCI subjects, of whom 133 were diagnosed with AD during follow-up, baseline brain age values demonstrated 81% accuracy in predicting conversion to AD during the first year of follow-up. This method was more accurate than predictions made based on chronological age, hippocampal volume, cognitive scores (MMSE), and CSF (cerebrospinal fluid) biomarkers (total tau, phospho-tau, Abeta42, and ratio of Abeta42/phospho-tau) (Gaser et al., 2013). The study also found that each additional brain age year was associated with a 10% elevated risk of developing AD (Gaser et al., 2013).

Investigations into correlations between brain age and neuropsychological and cognitive assessments have yielded varied results. Significant correlations have been reported between brain age and instruments including MMSE, CDR (Clinical Dementia Rating), and/or ADAS (Alzheimer’s Disease Assessment Scale) in AD subjects when pooled with healthy controls (Löwe et al., 2016; Beheshti et al., 2018; Wrigglesworth et al., 2021). Brain age gap also correlated with memory, attention, and language in amnestic MCI subjects and memory, executive function, and MMSE in MCI subjects (Huang et al., 2021). Significant correlation between brainAGE and MMSE in AD subjects, and between brainAGE and ADAS in progressive MCI subjects, have also been reported (Franke et al., 2012; Wrigglesworth et al., 2021). An MCI study also identified correlation between the brain age gap with CDR and ADAS at baseline; this correlation retained significance at follow-up at which point MMSE also demonstrated a correlation with brain age gap (Gaser et al., 2013; Wrigglesworth et al., 2021). Analysis of brain age across independent data sets of healthy adults have further found a negative relationship between brain age and cognitive function measures including general cognitive status, semantic verbal fluency (Franke et al., 2013), processing speed, and cognitive flexibility (Boyle et al., 2021). Lastly, in a cohort of healthy 45 year old adults, higher brainAGE correlated with cognitive decline and changes in IQ scores since childhood (Elliott et al., 2021).

Additional studies have developed alternative methodologies for estimating brain age. When using resting-state functional connectivity to determine brain age, although low accuracy of age prediction was observed, significantly increased brain age gaps were found in AD subjects compared to controls (Millar et al., 2022). Preclinical AD subjects also unexpectedly demonstrated younger brain ages compared to controls, although acceleration of brain age has been observed in preclinical AD (Gonneaud et al., 2021). While further studies are needed, decreased brain age in preclinical AD subjects may reflect compensatory responses to preclinical AD pathology or heterogeneity of brain network changes in aging and AD (Millar et al., 2022). It will thus be important to determine when, based on brain aging measures, a preventive intervention should be administered. Different interventions may have distinct mechanisms of action that may be more effective at different stages of disease progression, e.g., some interventions may ameliorate pathological processes occurring later in disease, whereas others may prevent progression at earlier stages of disease. Other studies have also evaluated the use of PET (positron emission tomography) imaging (Goyal et al., 2019), cortical thickness (Aycheh et al., 2018), and convolutional neural networks (Cole et al., 2017), which distinguished brain age gaps of early-onset (<65 years old) AD subjects from controls (Gautherot et al., 2021).

## Measuring biological age using DNA methylation profiling

Research into human longevity and “healthspan”—the average length of healthy years of life—has explored approaches toward measuring biological age, the level at which the body is biologically functioning, as compared against the expected level of functioning per chronological age (Rutledge et al., 2022). One approach is analysis of DNA methylation changes, e.g., cytosine-5 methylation of CpG (cytosine-phosphate-guanine) dinucleotides, to determine an individual’s “epigenetic clock” and predicted epigenetic age (DNAm) (Hannum et al., 2013; Horvath, 2013). These clocks may be used to identify changes in rates of biological aging and to investigate the impact of aging interventions (Belsky et al., 2020). The first generation of epigenetic clocks that were constructed include the Horvath clock (353 CpG sites) (Horvath, 2013) which was developed using 51 tissue types, and the Hannum DNAm score (71 CpG sites) (Hannum et al., 2013) which was developed using leukocytes. These measurements are predictive of chronological age (Horvath and Raj, 2018) and enables determination of biological age acceleration based on discrepancy between chronological age and DNAm age (Hannum et al., 2013; Horvath, 2013). The next generation of clocks incorporated clinical features and/or blood-based measurements to enable DNAm predictions of healthspan and mortality (McCrory et al., 2021); these include the DNAm PhenoAge (phenotypic age) score (513 CpG sites) (Levine et al., 2018) and the GRIMage score (1,030 CpG sites) (Lu et al., 2019). An increase in epigenetic age in relation to chronological age indicates age acceleration, which is related to adverse health outcomes (Fransquet et al., 2019; Oblak et al., 2021) and mortality (Oblak et al., 2021). Such acceleration has demonstrated associations with factors such as socio-economic status, gender, alcohol consumption, smoking status, and others (Ryan et al., 2020; Oblak et al., 2021). Estimation of age acceleration is further reflected in the development of a third generation DNA methylation biomarker, DunedinPACE (Pace of Aging Calculated from the Epigenome) (Belsky et al., 2022). This biomarker was constructed using the Dunedin Longitudinal Study cohort for whom 19 biomarkers of multiple organ systems (cardiovascular, metabolic, renal, hepatic, immune, periodontal, and pulmonary) were measured longitudinally in subjects at 26, 32, 38, and 45 years of age. The resulting pace of aging model from this data was distilled into a single DNA methylation biomarker that captures cross-system aging-related changes.

## Biological age in the context of cognitive functions and Alzheimer’s disease

Epigenetic age has been found to be associated with AD (Table 2). The Horvath clock and PhenoAge score are associated with neuropathological markers of AD, including diffuse and neuritic plaques, amyloid load, and neurofibrillary tangles, based on analysis of AD prefrontal cortex (Levine et al., 2015, 2018). The PhenoAge score was found to also predict all-cause mortality, age-related morbidity, healthspan, and AD, along with physical function measures (Levine et al., 2018). Additionally, the Horvath clock was a significant predictor of dementia in a late-middle-aged cohort (55–65 years) with 15 years of follow-up (Degerman et al., 2017); and demonstrated an association with ante-mortem cognitive and memory decline in AD, although no correlation between AD dementia status and DNAm was identified (Levine et al., 2015). The absence of this correlation may reflect the underlying complexity of AD pathogenesis, which was exemplified by the high level of inter-individual variability of cognitive deficits in AD subjects (Levine et al., 2015).

**TABLE 2:** Human epigenetic age studies on cognitive functions, dementia, mild cognitive impairment (MCI), and/or Alzheimer’s disease (AD).

References	Study focus	Cohorts	No. of subjects (female)	Mean age ± SD	Analyzed tissue	Measurements	Primary findings
Levine et al., 2015	To examine association between epigenetic age and cognitive decline and neuropathological markers of AD	ROS, MAP	700 (445)	81.36 ± 6.59	Dorsolateral prefrontal cortex (DLPFC)	DNAm (Horvath); cognitive test battery; neuropathological assessments	Acceleration of epigenetic age was associated with diffuse and neuritic plaques, amyloid load, global cognitive functioning, and episodic and working memory in AD subjects. Acceleration was heritable and demonstrates genetic correlation with diffuse plaques and possibly working memory.
Degerman et al., 2017	To evaluate epigenetic age changes in groups with different memory trajectories over a 15 year period	Betula Longitudinal Study	52 (25)	57.90 ± 0.10 (baseline); 72.77 ± 0.06 (follow-up)	Peripheral blood	DNAm (Horvath); episodic memory tests	DNAm age differed significantly across high/average/low memory groups with the high memory group (who maintained memory functions) showing younger DNAm age by 2.7–2.8 years. DNAm at follow-up, and not chronological age, was a predictor for dementia (AD or vascular dementia).
Levine et al., 2018	To develop and assess a new epigenetic biomarker of aging, PhenoAge	ROS, MAP	700 (445)	81.36 ± 6.59	DLPFC	DNAm (PhenoAge)	Age-adjusted PhenoAge was positively associated with AD neuropathologies (amyloid load, neuritic plaques, neurofibrillary tangles). PhenoAge is higher in AD subjects versus controls.
Beydoun et al., 2020	To assess sex-specific relationships of DNAm with cognitive performance in middle-aged subjects	HANDLS	199 (95)	56.14 ± 0.36	Blood mononuclear cells	DNAm (Horvath, Hannum); cognitive performance batteries	Acceleration of DNAm (Hannum) was related to increased cognitive decline in men per visual memory and attention domains.
Sibbett et al., 2020	To assess if DNAm age acceleration is associated with risk of dementia	LBC1921	488 (280)	79.06 ± 0.03	Whole blood	DNAm (Horvath, GrimAge, PhenoAge); intelligence and cognitive test batteries; APOE genotype	No association was found between increased epigenetic age acceleration and dementia risk.
Grodstein et al., 2021	To determine if DNAm in cortex samples from older subjects are related to clinical and neuropathological measures of AD	ROS, MAP	721 (461)	88.00 ± 6.70	DLPFC	DNAm (Horvath, Hannum, PhenoAge, GrimAge, cortical)	All DNAm clocks were associated with Abeta load and AD pathological diagnoses with the cortical clock demonstrating the strongest associations. The cortical clock was the only one significantly associated with mean tangle density and measures of cognitive decline.
Grodstein et al., 2020	To evaluate how blood-based DNAm measurements compare to brain tissue-based DNAm in older subjects and in the context of AD diagnoses	ROS, MAP	(1) 41 (27); (2) 730 (467); (3) 186 (122)	(1) 88.70 ± 4.70; (2) 88.00 ± 6.70; (3) 90.00 ± 6.00	(1) Peripheral blood CD4 ± cells; (2) DLPFC; (3) posterior cingulate cortex	DNAm (Horvath, Hannum, PhenoAge, cortical)	All clocks were lower than chronological age. Epigenetic age was modestly correlated across blood and DLPFC samples from the same subjects. When stratified by sex, AD pathology diagnosis, and AD clinical diagnosis, correlations between epigenetic and chronologic ages were high.
Fransquet et al., 2021	To determine if acceleration of epigenetic clocks predicts dementia risk	ASPREE	160 (92)	77.00 ± 0.85	Peripheral blood	DNAm (Horvath, Hannum, PhenoAge, GrimAge); cognitive test battery	Subjects with pre-symptomatic dementia did not demonstrate accelerated epigenetic aging compared to controls.
Vaccarino et al., 2021	To assess if DNAm age acceleration is associated with cognitive decline in cognitively healthy twins	VET registry: ETS	266 twins [133 pairs] (0)	56.00 ± 3.00	Whole blood (lymphocytes)	DNAm (Horvath, Hannum, Pheno Age, Grim Age); cognitive test battery	At baseline, there was no relationship between DNAm acceleration and cognitive functions. In longitudinal analyses of intra-twin analyses, each additional year of age (Horvath) was related to a 3 and 2.5% decrease in the executive function and memory function scores, respectively. Middle-aged men with higher DNAm relative to their brother showed a faster rate of cognitive decline based on 11.5 year follow-up.
Sugden et al., 2022	To assess blood-based DNA methylation measures of biological aging in relation to cognitive functions and clinical diagnoses in cognitively normal, MCI, and AD subjects; to assess if DNA methylation measures of biological aging predict dementia risk	ADNI, FHS	ADNI: 649 (288); FHS: 2,264 (NA)	ADNI: 74.77 ± 7.66; FHS: NA	ADNI: whole blood; FHS: buffy coat	DNAm (Horvath, Hannum, PhenoAge, GrimAge); DunedinPACE; cognitive test batteries	Only DunedinPACE was associated with clinical diagnosis of AD and cognitive impairment in the ADNI cohort. DunedinPACE was associated with risk of dementia in the FHS cohort.

ROS, Religious Orders Study; MAP, Rush Memory and Aging Project; VET, Vietnam Era Twin; ETS, Emory Twin Study; ASPREE, ASPirin in Reducing Events in the Elderly; LBC1921, Lothian Birth Cohort 1921; HANDLS, Healthy Aging in Neighborhoods of Diversity across the Lifespan; ADNI, Alzheimer’s Disease Neuroimaging Initiative; FHS, Framingham Heart Study.

Analysis of epigenetic age acceleration has yielded variable findings in middle-aged adults and broadly with respect to dementia risk (Table 2). In studies of middle-aged adults, no relationship between epigenetic age, or age acceleration, with cognitive function was observed (Starnawska et al., 2017; Belsky et al., 2018; Vaccarino et al., 2021). However, epigenetic age acceleration was associated with increased cognitive decline in middle-aged men within visual memory and attention domains (Beydoun et al., 2020). In terms of dementia risk, in a study of older adults with pre-symptomatic dementia compared to controls (mean age 77.0 years), accelerated epigenetic aging did not predict dementia risk (Fransquet et al., 2021). Another study also evaluated epigenetic age acceleration in incidental dementia, and adjusted for multiple covariates including APOE genotype, age, and sex, but did not observe a relationship between age acceleration and dementia incidence (Sibbett et al., 2020). However, these studies did not differentiate between AD and other types of dementia, which may harbor different underlying pathological processes, and the former study did not adjust for APOE genotype. Even so, age acceleration associated with AD has been observed in an AD mouse model (Coninx et al., 2020), and the Hannum and PhenoAge clocks both demonstrated an association between epigenetic age acceleration and hippocampal volume in AD (Milicic et al., 2022).

Such variability in findings may be associated with heterogeneity in sampled populations and aging processes within and across subjects, and factors associated with measurement of the epigenetic clock, such as the use of variable training sets, tissues, and algorithms. The absence of correlations may also arise from capturing distinct aging processes. Inter-individual variability in methylation profiles, based on the Horvath clock, has been observed in those with the same chronological age and different epigenetic age, as well as for those with the same chronologic and epigenetic age (Shahal et al., 2022). In middle-aged adults, Horvath’s clock also demonstrated variability within separate memory performance groups, with a lower epigenetic age observed in subjects who sustained memory functions over time (Degerman et al., 2017). Horvath’s clock may also exhibit bias in older individuals (>60 years) and when analyzing different types of tissues (El Khoury et al., 2019). Interestingly, multiple studies have observed that older individuals, e.g., >70 years, demonstrate younger epigenetic ages compared to chronological age (Degerman et al., 2017; El Khoury et al., 2019; Fransquet et al., 2021) and that age acceleration slows or reverses in older subjects (El Khoury et al., 2019; Fransquet et al., 2021).

Recent studies have expanded analysis to human brain tissue to develop DNAm methods for age prediction. Shireby et al. (2020) developed an epigenetic clock, DNAmClockCortical (347 CpG sites), using human cortex DNA methylation data collected across a wide age range (1–108 years; *n* = 1,397) with exclusion of AD subjects. This clock was validated in an independent human cortex data set, was found to be associated with lower neuron proportions, and more accurately predicted age of brain tissue samples compared to the Horvath and PhenoAge clocks, as well as compared to a third blood and saliva-based clock (Zhang et al., 2019; Shireby et al., 2020). In an analysis of both blood and prefrontal cortex from the same subjects, modest correlation of epigenetic age (Horvath, Hannum, PhenoAge, and DNAmClockCortical) was observed with DNAmClockCortical outperforming the other clocks (Grodstein et al., 2020). However, a separate analysis found that while DNAmClockCortical showed high predictive ability, there was under-estimation of DNAm age in subjects > 60 years of age and a non-linear relationship was found between DNAm and chronological age (Shireby et al., 2020).

In an independent analysis, Grodstein et al. (2021) evaluated the performance of multiple epigenetic clocks, including the Horvath, Hannum, PhenoAge, GrimAge, and DNAmClockCortical measures, on prefrontal cortex from older subjects (mean age at death of 88.0 + /6.7 years; *n* = 721). Within this cohort, almost half of the participants had a diagnosis of dementia, including AD dementia. Overall, all clocks, except for GrimAge, showed significant association with global AD pathology, encompassing neuritic and diffuse plaque load and neurofibrillary tangle burden, with DNAmClockCortical demonstrating the greatest association (*P* < 0.00001). This was reflected in the finding that for each standard deviation increase in DNAmClockCortical age, there was a 90% increased probability of pathologic AD (odds ratio = 1.91, 95% confidence interval 1.38, 2.62), compared to a 30% increased probability when using the Hannum, Horvath, or PhenoAge clocks (no relation was identified for GrimAge). In analyses of the epigenetic clocks in relation to clinical and genetic factors, including dementia and AD dementia diagnoses, cognition and memory scores, and APOE genotype, it was found that older epigenetic ages based on the Hannum, Horvath, and PhenoAge clocks were not associated with increased likelihood of AD dementia, global cognitive decline over time, or specific cognitive function declines. In contrast, DNAmClockCortical was significantly associated with all these measures, and older epigenetic age was also associated with APOEε4 carrier status. This study underscores the unique nature of brain tissue when estimating epigenetic age, how epigenetic clocks may need to be tailored to disease processes, and that utilizing CpG sites that are conserved in both brain and other tissues will be important to explore (Grodstein et al., 2021). It further highlights the need to consider other biological aging clocks that are not based on epigenetic phenomena and that utilize other tools such as transcriptomics, proteomics, and metabolomics (Rutledge et al., 2022).

## Integrating brain and biological age to assess aging trajectories

Estimation of brain and biological age gaps and acceleration have shown promise as potential biomarkers for identifying changes in aging trajectories and for use in mid-life intervention studies aimed at preventing MCI and AD. One challenge with determining biological age in the context of brain health is sample access. As a result, developing ante-mortem approaches, such as using peripheral blood, is needed. Moreover, since AD is also associated with other health factors that may influence biological aging, the use of integrated biomarker strategies that consider both brain and biological ages may be beneficial. One example of implementing such an approach was the evaluation of brainAge in the previously described Dunedin Longitudinal Study cohort of middle-aged subjects (45 years), in combination with the “pace of aging” biomarker of biological age (Belsky et al., 2015). This analysis was based on the previously mentioned 19 pace-of-aging biomarkers, and cognitive functions, from longitudinal data collected between 3 and 45 years of age for each subject (Elliott et al., 2021). Compared to individuals of the same chronological age but younger brainAge, individuals with older brainAge demonstrated accelerated pace of biological aging between 26 and 45 years of age; reduced adult cognitive functions; and rapid facial aging. They also demonstrated decreased childhood cognitive functions and accelerated cognitive aging. This study provides evidence that older mid-life brainAge is related to early life differences and that accelerated aging may accumulate during life (Elliott et al., 2021). Thus, brain and biological age measures may capture overlapping aging features and may be influenced by individual differences that occur through-out life.

The DunedinPACE biomarker, which reflects aging-related changes across organ systems, also represents one solution toward capturing such overlapping features. DunedinPACE was developed using the DunedinPoAm (Dunedin[P]lace[o]f[A]ging[m]ethylation) algorithm which takes advantage of blood-based DNA methylation patterns that are associated with 19 pace-of-aging biomarkers (Belsky et al., 2020, 2022). In a cohort of cognitively normal older adults and subjects with MCI or AD, a comparison of different generations of epigenetic age measurements (Horvath and Hannum clocks, PhenoAge and GrimAge, DunedinPACE), DunedinPACE was the only measure that was associated with a diagnosis of AD and worse cognitive function scores (Sugden et al., 2022). Increased pace-of-aging, based on DunedinPACE, was also found to be associated with risk of developing dementia in a separate cohort (Sugden et al., 2022).

## Discussion

Although brain and biological age measures have yet to be widely adopted into clinical trials, their continued development and evolution have yielded promising biomarkers to evaluate in future studies designed to assess the efficacy of geroprotectors and other interventions for treating MCI and AD. This lack of adoption is associated with multiple factors including: (1) evaluation of AD interventions has historically focused on identifying changes in disease symptoms including cognitive functions and/or neuroimaging alterations, rather than divergence away from healthy aging trajectories.; (2) more research is needed to determine if measuring this divergence is both clinically actionable and meaningful.; (3) Studies are also needed to evaluate if “improving” brain and biological age biomarkers is associated with actual positive brain and health changes, and if these changes result in slowing down the progression of, or preventing, onset of AD.; (4) particularly for biological age measures, it remains unclear as to how specific this estimate is for portraying brain health. For example, can someone who is neurologically healthy demonstrate an advanced biological age primarily due to accelerated aging in other organ systems?; and (5) the cost of collecting additional measurements in clinical trials may currently be prohibitive, especially since the clinical utility of brain and biological ages is still unclear. Even so, as future studies continue to shed light on these questions and optimize strategies for measuring brain and biological ages, the creation of standardized and validated approaches for determining brain and biological age will ultimately enable us to test if interventions truly slow aging and if this change further slows development of, or prevents onset of, MCI, AD, and other neurodegenerative diseases.

Based on existing brain and biological aging research, one penultimate phenomenon that has become common knowledge is the unique nature of aging trajectories in the brain and body for each person (Vieira et al., 2017; Aycheh et al., 2018; Elliott et al., 2021) (i.e., pronounced inter-individual variation in aging rates). This is embodied both by the “early system integrity” concept for which varied aging trajectories in the brain and body begin at birth and are influenced by genetic, lifestyle, and environmental factors (Deary, 2012; Elliott et al., 2021), as well as the geroscience perspective for which age-related decline occurs across multiple body systems (Kennedy et al., 2014; Elliott et al., 2021). Importantly, both brain age and biological age measures demonstrate non-linear changes at older ages (Huang et al., 2021), such as in long-lived supercentenarians (Horvath et al., 2015; Armstrong et al., 2017). The complexity and heterogeneity of these measurements highlight the utility of performing single subject (N-of-1) longitudinal analyses (Lillie et al., 2011; Schork and Goetz, 2017) to identify important health shifts in aging. Currently, biological age estimation can be performed with relative ease from blood collections, whereas brain age measurements require neuroimaging solutions. However, further evaluating multi-system measures such as DunedinPACE and estimating both brain and biological ages, provide complementary data that may be valuable for identifying interventional opportunities aimed at preventing or slowing onset of AD.

## Author contributions

WL drafted the manuscript. All authors contributed to manuscript revision, read, and approved the submitted version.
